# Disparities in lung cancer case registration, treatment, and mortality: descriptive study, Brazil, 2013-2019

**DOI:** 10.1590/S2237-96222026v35e20240909.en

**Published:** 2026-04-12

**Authors:** Lara Vinhal Faria, Isabel Martins Emmerick, Monica Rodrigues Campos

**Affiliations:** 1 Fundação Oswaldo Cruz, Escola Nacional de Saúde Pública Sérgio Arouca, Rio de Janeiro, RJ, Brazil Fundação Oswaldo Cruz Escola Nacional de Saúde Pública Sérgio Arouca Rio de Janeiro RJ Brazil; 2 Division of Thoracic Surgery, Department of Surgery, UMass Chan Medical School, Worcester, MA, United States Division of Thoracic Surgery Department of Surgery UMass Chan Medical School Worcester MA United States

**Keywords:** Lung Neoplasms, Mortality Registration, Hospital Cancer Registries, Socioeconomic Health Disparities, Retrospective Studies., Neoplasias Pulmonares, Registros de Mortalidad, Sistemas de Información en Salud, Disparidades en el Estado de Salud, Estudios Retrospectivos.

## Abstract

**Objective:**

To analyze factors associated with lung cancer case registration, treatment and mortality in Brazil, 2013-2019.

**Methods:**

This was a retrospective study using the Brazilian Mortality Information System (*Sistema de Informação de Mortalidade* - SIM) and the Hospital Cancer Registries (*Registros Hospitalares de Câncer* - RHC). Outcomes analyzed were: (Model 1) "absence of lung cancer case registration" in the RHC, and (Model 2) "rate of registered cases above the 3rd quartile." Descriptive and bivariate analyses were performed to compare groups, as well as multivariate logistic regression, identifying, at the municipal level, factors associated with the outcomes. Adjusted odds ratios (OR) and 95% confidence intervals (95%CI) were obtained.

**Results:**

A total of 206,703 deaths and 89,265 cases (43% coverage) were analyzed. Approximately 85.7% of patients were diagnosed at a late stage, and half of the cases with initial staging did not receive adequate treatment. In Model 1, a lack of RHC coverage was associated with municipalities in the Central-West region compared to those in the North [OR 3.9; 95%CI 2.7; 5.7] and with those having a Gross Domestic Product below the national average [OR 1.3; 95%CI 1.1; 1.7]. In Model 2, municipalities in the South [OR 9.1; 95% CI 6.8; 12.2], small-sized municipalities [OR 7.1; 95%CI 4.0; 12.3], and those with below-average travel distances for treatment [OR 4.2; 95%CI 2.3; 7.7] had increased odds of a high registration rate.

**Conclusion:**

Localities with better infrastructure were more likely to register cases and have greater coverage of registries, while areas with poorer infrastructure were more likely not to register cases, despite presenting deaths due to lung cancer.

## Resumen

**Objetivo:**

Analizar los factores asociados al acceso al registro de casos, a las características del tratamiento y a la mortalidad por cáncer de pulmón entre 2013 y 2019 en Brasil.

**Métodos:**

Estudio retrospectivo utilizando el Sistema de Información sobre Mortalidad (SIM) y los Registros Hospitalarios de Cáncer (RHC). Se analizaron los siguientes desenlaces: (Modelo 1) "ausencia de registro de caso de cáncer de pulmón" en el RHC y (Modelo 2) "tasa de casos registrados por encima del 3er cuartil". Se realizaron análisis descriptivos y bivariados para comparar los grupos, y regresión logística multivariada, identificándose, a nivel municipal, los factores asociados a los desenlaces, obteniéndose la razón de momios ajustada (odds ratio, OR) y el intervalo de confianza del 95% (IC95%).

**Resultados:**

Se analizaron 206.703 defunciones y 89.265 casos (43% de cobertura). Aproximadamente 85,7% de los pacientes fueron diagnosticados en etapas avanzadas, y la mitad de los casos con estadificación inicial no recibió el tratamiento adecuado. En el Modelo 1, la falta de cobertura del RHC se asoció con municipios de la región Centro-Oeste en comparación con la Norte [OR 3,9; IC95% 2,7; 5,7] y con producto interno bruto por debajo del promedio nacional [OR 1,3; IC95% 1,1; 1,7]. En el Modelo 2, la mayor probabilidad de una tasa alta de registro se observó en municipios del Sur [OR 9,1; IC95% 6,8; 12,2], de pequeño porte [OR 7,1; IC95% 4,0; 12,3] y con desplazamiento para tratamiento por debajo del promedio nacional [OR 4,2; IC95% 2,3; 7,7].

**Conclusión:**

Localidades con mejor infraestructura presentaron mayor probabilidad de registrar casos y tener mayor cobertura de registros, mientras que áreas con menor infraestructura presentaron mayor probabilidad de no registrar casos, a pesar de registrar defunciones por cáncer de pulmón.

**Ethical aspects**

This research used public domain anonymized databases.

## Introduction 

According to global estimates for 2022, lung cancer is the most lethal, with 2.2 million new cases and 1.8 million deaths per year ^1^. In Brazil, it is estimated that there will be 30,000 new cases annually during the 2023-2025 triennium ^2^.

Smoking is a significant risk factor for developing the disease, with approximately 85% of cases associated with tobacco use. For this reason, Brazil's main public policy aimed at reducing lung cancer mortality consists of anti-smoking initiatives ^3^.

Overall, diagnosis occurs at advanced stages, and survival rates are low. Nearly 85% of patients present with locally advanced or metastatic disease (stages III and IV, respectively) at the time of diagnosis ^4^. Lung cancer has nonspecific symptoms, which makes early-stage diagnosis particularly challenging ^5^. This issue highlights barriers to accessing oncology services and reveals shortcomings in their availability and quality ^6^.

Currently, there is a movement toward implementing lung cancer screening in Brazil, since this would be a viable strategy for detecting the disease at earlier stages and, consequently, reducing mortality ^7^.

The distribution of imaging equipment and specialized treatment centers is uneven across the country, which has consequences for the health outcomes of individuals living in different locations ^8^. Evidence from the international literature shows that unfavorable sociodemographic and economic characteristics are associated with lower survival rates and a worse prognosis ^9^. However, in Brazil, knowledge on this topic remains scarce.

Thus, the objective of this study was to analyze access to case registration, treatment characteristics, and mortality due to lung cancer between 2013 and 2019, investigating associated factors.

## Methods 

Study Design

This was a retrospective study using secondary national databases: censuses from the Brazilian Institute of Geography and Statistics (*Instituto Brasileiro de Geografia e Estatística* - IBGE), data from the United Nations Development Programme (UNDP), the Brazilian Mortality Information System (*Sistema de Informações sobre Mortalidade* - SIM), and the Hospital Cancer Registries (*Registros Hospitalares de Câncer* - RHC). Data triangulation was conducted at the municipal level, utilizing the IBGE identification code. Lung cancer cases from the RHC were selected based on place of residence and identified using code C34 of the International Statistical Classification of Diseases and Related Health Problems 10th Revision (ICD-10). Analyses were stratified by population size and macroregion: small (<100,000 inhabitants), medium (100,000-400,000 inhabitants), and large (>400,000 inhabitants).

Variables

1) Sociodemographic indicators, estimated from the 2010 demographic census - IBGE and UNDP: gross domestic product (GDP) per capita in 2019; education level (percentage of people aged 25 years or more with up to 8 years of schooling); urbanization (percentage of the municipal population living in urban areas); life expectancy.

For supplementary health coverage, data from the Brazilian National Agency for Supplementary Health were used, which reflect the percentage of the population covered by private health plans, as of June 2019.

Primary health care coverage was obtained from the e-Gestor AB system, and supplementary health coverage was obtained from the Brazilian Beneficiary Information System. The number of physicians per 1,000 inhabitants was also obtained from the Brazilian Beneficiary Information System.

2) Lung cancer cases registered in the RHC between 2013 and 2019 and clinical indicators: advanced stage (percentage of individuals with stages III and IV); Missed opportunity instead of loss (percentage of patients with early stage who did not undergo surgery or radiotherapy); timely treatment (percentage of individuals who began treatment within 60 days after diagnosis) ^10^; commuting (percentage of patients treated outside their municipality of residence); age; percentage of patients: White, with higher education, diagnosed without treatment, undiagnosed and untreated, and treated. 

For the calculation of timely treatment, the most favorable criterion was adopted to define the date of first contact with the service (date of first consultation or date of screening test, whichever was earliest). Patients not eligible for treatment due to a lack of therapeutic possibility were excluded from this analysis.

3) Characterization of lung cancer deaths recorded in the SIM from 2013 to 2019, with calculation of lung cancer mortality rate per 100,000 inhabitants: mean age; percentage of male deaths; percentage of deaths among individuals with higher education.

The period from 2013 to 2019 was chosen due to the consistency and high data quality in the RHC, and to avoid the impact of the COVID-19 pandemic on both health services and care-seeking patterns ^11^.

Correction methods for both SIM and RHC have been detailed in a previous article ^4^.

Statistical methods

To identify factors associated with disparities in case and death registration, the outcome "absence of lung cancer case registration" in the RHC was analyzed using a multivariate logistic regression model (Model 1). To characterize access to treatment and aspects of its quality in the RHC, after sensitivity analysis, the outcome "rate of registered lung cancer cases above the 3rd quartile" was selected for the period 2013-2019, based on the estimated 2019 population and municipality of residence, using multivariate logistic regression (Model 2).

In the descriptive and bivariate analyses, exposure variables were characterized by their mean, standard deviation, minimum, and maximum values, according to the categories of each outcome.

For continuous exposure variables, Student's t-test was applied (p-value<0.05), using SPSS v21, followed by dichotomization into "0" and "1." 

The reference category for each variable was based on the national average: if the variable was higher in category "1" of the outcome, it was coded as "above the national average." All exposure variables, therefore, had "above the national average" as the reference category, except for the percentage of White lung cancer patients (RHC), mean age (RHC), percentage of patients with higher education (RHC), mean age (SIM), and primary health care coverage. Unadjusted odds ratios (OR) and 95% confidence intervals (95%CI) were presented, indicating increased odds compared to the reference value.

During model construction, coverage rates were initially targeted. After iterative sensitivity analyses, two final models were obtained, adjusted for statistically significant variables within the theoretical explanatory model of access. Thus, adjusted ORs were estimated: in Model 1 using the enter method, and in Model 2 using stepwise backward selection.

Databases were processed using Python v3.7, and statistical analyses were conducted using SPSS v21.

**Figure 1:**
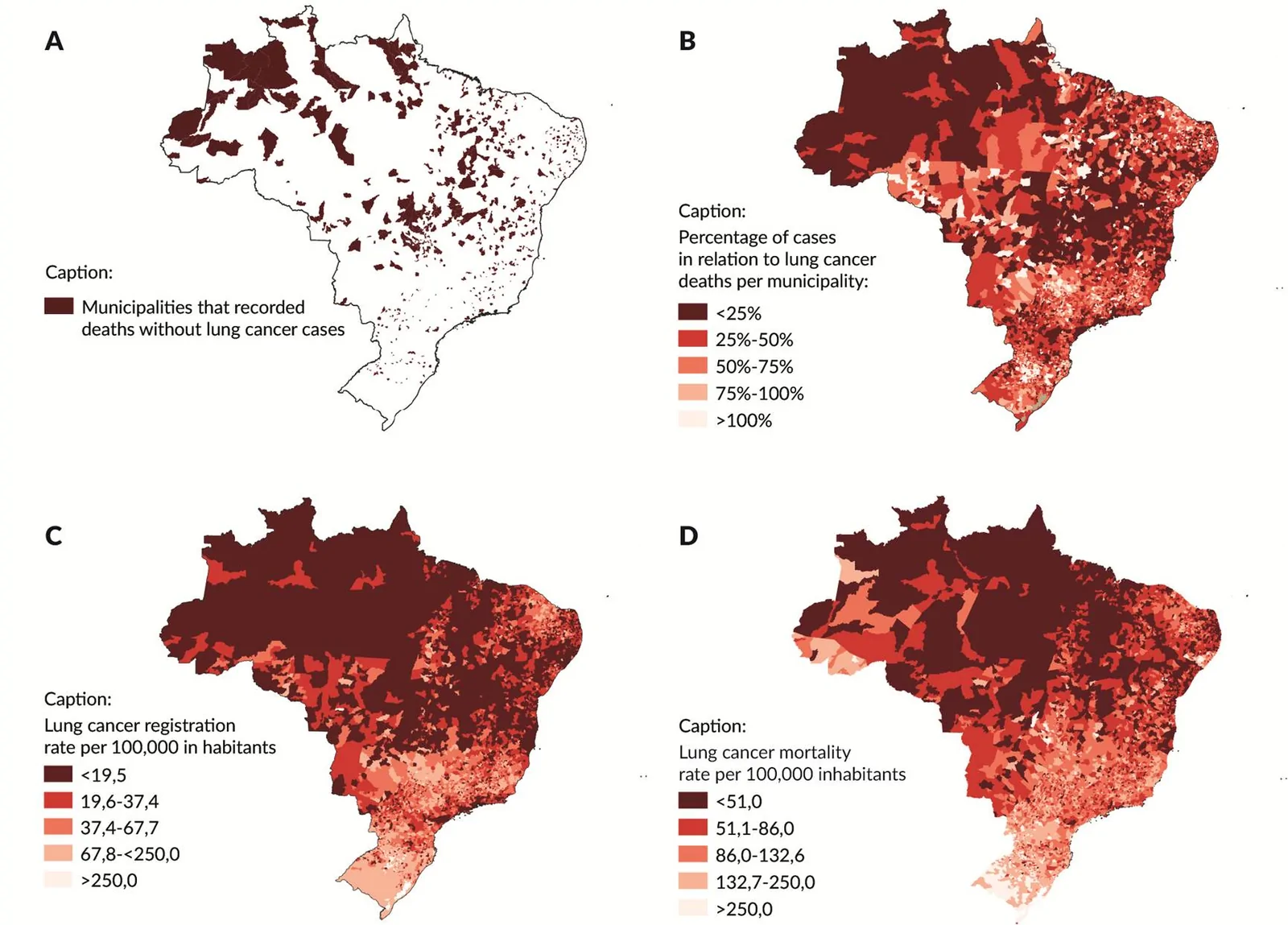
Municipalities with no recorded lung cancer deaths in the Mortality Information System (SIM) (A); number of cases recorded in the Hospital Cancer Registry (RHC) relative to the number of lung cancer deaths in the SIM (B); lung cancer registration rate per 100,000 inhabitants (RHC) (C); lung cancer mortality rate per 100,000 inhabitants (SIM) (D). Brazil, 2013-2019

## Results 

After correction, 206,703 deaths from lung cancer were recorded in Brazil between 2013 and 2019, with a mortality rate of 99.4 per 100,000 inhabitants. The highest mortality rate was observed in the South region (161.7 per 100,000), nearly three times higher than that of the North (54.8 per 100,000), the lowest in the country (Figure 1).

Correction percentages in the SIM were: 0.03% for sex, 2% for age, 2% for garbage codes, 6% for ill-defined causes, and 4.7% for underreporting. Although small, these corrections were important for improving the completeness of information; corrections to staging in the RHC were crucial, preventing 32% of data loss.

Sociodemographic indicators by region and population size showed pronounced disparities and gradients within each region. The average GDP per capita of large municipalities in the North and Northeast was equivalent to that of small municipalities in other regions, revealing a wide disparity in financial, human, and infrastructure resources. In terms of health indicators, primary health care coverage ranged from 61.4% in large municipalities to 93.7% in small ones, while supplementary health coverage was inversely distributed (Table 1).

During the same period, 89,265 lung cancer cases were recorded in the RHC, with over 80% of patients starting treatment within 60 days of diagnosis; however, the majority (85.7%) had advanced-stage disease. In the North, more than 50% of patients with early-stage disease did not undergo surgery or radiotherapy as first treatment (Table 2).

The RHC recorded approximately 43% coverage of lung cancer cases compared to SIM deaths ("RHC/SIM coverage"), evidencing a significant gap between registered cases and lung cancer deaths.

The Central-West region, particularly Goiás, had the highest number of municipalities with recorded lung cancer deaths but no cases registered in the RHC. States with the lowest "RHC/SIM coverage" (<25%) were Goiás, Amazonas, and Acre. Regarding registration rates in the RHC and mortality in the SIM, municipalities with higher rates were mainly located in the South and Southeast regions (Figure 1).

In the bivariate analysis, municipalities with "absence of case registration" (Model 1 outcome) presented worse sociodemographic and health indicators. In contrast, those with lung cancer registration rates above the 3rd quartile (Model 2 outcome) had higher sociodemographic indicators compared to the others, in addition to lower percentages of advanced-stage disease, displacement, and patients diagnosed but untreated (Table 3).

In the multivariate logistic regression (Model 1), municipalities in the Central-West region, compared to those in the North, were more likely to have no lung cancer cases registered in the RHC. This outcome was also associated with municipalities that had a higher percentage of deaths outside the Brazilian National Health System (SUS) (private sector), a percentage of individuals with higher education (SIM) below the national average, and primary health care coverage above the national average (Table 4).

In Model 2, variables associated with higher odds of the outcome were: residing in the South region; being a small municipality; and commuting below the national average. One of the highest adjusted ORs observed in Model 2 was for the variable "intermunicipal commuting for treatment," with municipalities being 4.2 times more likely to have high registration rates when displacement percentages were below the national average (Table 4). 

White individuals, those with higher education, older adults, and those living in localities with better infrastructure for diagnosis and treatment had higher odds of lung cancer cases being registered above the 3rd quartile (Table 4).

**Table 1:** Distribution of lung cancer deaths and sociodemographic indicators by region and municipal size. Brazil, 2013-2019

Municipal Size	Total Deaths	Mortality Rate/100,000 inhabitants	Male (%)	Higher education (%)	Mean age	GDP per capita	Education level (%)	Life expectancy at birth	HDI^a^	Urbanization (%)	Physicians/1,000 inhabitants	Primary health care coverage (%)	Supplementary health coverage (%)	Deaths outside SUS (%)
Brazil	206703	99.4	57.9	7.5	68.1	24539.90	48.9	73.06	0.66	63.8	2.0	92.2	22.5	11.1
Small	83420	94.4	60.3	3.1	68.3	23730.38	47.8	72.93	0.65	62.0	0.9	93.7	9.5	3.9
Medium	46825	99.3	57.7	6.1	67.4	36631.09	64.7	75.09	0.73	91.9	1.9	69.9	24.1	9.6
Large	76458	105.5	55.5	13.1	68.3	41842.99	72.4	75.45	0.77	98.0	3.2	61.4	37.4	19.9
North	9869	54.8	60.0	4.7	67.6	18415.07	47.4	71.82	0.61	56.7	1.0	87.5	9.4	7.9
Small	4008	46.5	62.3	2.2	67.4	17911.74	46.5	71.73	0.60	55.0	0.5	88.9	2.1	4.3
Medium	1649	43.1	62.4	3.5	67.4	25369.85	57.8	72.82	0.66	76.5	1.1	70.6	8.6	5.6
Large	4212	75.6	57.0	7.5	67.8	25828.52	71.3	74.05	0.73	96.2	1.8	62.7	21.4	12.2
Northeast	42424	75.0	53.9	5.2	68.5	12584.70	40.1	70.17	0.59	55.2	1.3	97.0	11.6	4.5
Small	22567	68.4	55.0	1.7	68.9	12221.96	39.4	70.06	0.59	54.0	0.6	97.6	2.8	1.6
Medium	7138	76.0	53.0	4.8	68.3	21850.04	57.4	72.88	0.68	86.1	1.5	83.7	14.4	4.1
Large	12719	89.9	52.5	11.6	67.8	25502.54	68.6	74.33	0.75	97.8	2.8	65.9	30.4	9.9
Southeast	92831	105.9	57.9	9.8	68.1	27816.66	54.4	74.68	0.70	74.9	2.5	88.9	32.5	16.7
Small	26285	103.5	60.8	4.4	68.2	26197.51	52.8	74.56	0.69	72.7	1.2	91.7	18.1	5.5
Medium	22040	100.1	58.6	7.4	67.3	42383.80	68.5	75.95	0.76	96.1	2.2	62.8	31.5	13.5
Large	44506	110.6	55.9	14.1	68.5	48688.28	73.0	75.84	0.78	98.5	3.4	58.7	42.2	24.8
South	48079	161.7	60.7	5.5	68.0	36247.70	53.4	75.12	0.71	60.7	2.2	91.2	23.2	6.3
Small	25620	170.4	63.5	3.1	68.1	35843.55	52.7	75.05	0.71	59.1	1.2	92.1	13.9	3.8
Medium	13652	156.0	58.3	5.4	67.2	44284.70	67.3	76.42	0.76	94.1	2.3	74.2	25.1	7.0
Large	8807	147.8	56.0	12.8	68.9	49135.75	76.6	76.61	0.81	97.8	4.5	71.3	43.9	12.4
Central-West	13500	84.1	59.3	8.0	67.4	34780.18	52.9	74.35	0.69	71.9	2.0	92.9	20.1	13.4
Small	4940	78.3	62.8	2.6	67.6	34640.97	52.2	74.30	0.69	70.6	0.9	94.5	10.6	6.7
Medium	2346	73.9	57.8	4.3	67.3	35365.75	63.1	75.15	0.73	94.6	1.5	67.6	18.0	6.9
Large	6214	94.6	57.0	13.7	67.4	44888.69	72.8	75.65	0.78	98.5	3.3	52.2	30.4	21.2

a Human development index.

**Table 2:** Distribution of lung cancer cases, case registration rates, and clinical and sociodemographic indicators by region and municipal size. Brazil, 2013-2019

Municipal Size	Total cases	Incidence rate per 100,000 inhabitants	Mean age	Male patients (%)	White patients (%)	Patients with higher education (%)	Advanced staging (%)	Timely care (%)	Lost opportunity n (%)	Commuting (%)	Without diagnosis and treatment (%)	With diagnosis but without treatment (%)	Treated patients (%)
Brazil	89265	44.2	64.4	56.5	37.1	6.1	85.7	82.7	5,166 (40.5)	56.0	46.1	40.7	70.1
Small	34878	42.1	64.2	60.1	42.5	3.7	86.3	83.3	1,984 (41.6)	97.5	47.3	41.6	70.8
Medium	20596	43.8	64.1	56.1	43.4	6.0	85.6	83.1	1,231 (41.6)	54.4	43.3	41.8	71.7
Large	33791	47.0	64.7	53.1	27.7	8.6	85.1	81.8	1,951 (38.7)	14.1	46.5	39.1	68.3
North	3470	20.8	63.7	58.9	14.1	4.5	86.9	84.8	254 (55.9)	54.7	39.1	50.7	67.8
Small	1291	17.8	63.1	59.5	17.4	2.5	86.1	85.1	100 (55.9)	97.2	38.1	51.4	72.3
Medium	703	18.4	64.7	59.6	13.2	5.5	87.6	83.9	50 (57.5)	59.9	45.0	46.1	73.7
Large	1476	26.5	63.8	58.0	11.6	5.8	87.3	85.0	104 (55.3)	15.0	37.2	52.3	60.9
Northeast	18542	34.3	64.2	52.1	14.2	4.5	88.2	85.5	1,012 (46.3)	54.6	34.2	52.4	59.0
Small	7593	24.9	63.8	54.6	12.1	1.7	87.8	85.1	429 (46.4)	99.4	35.7	53.3	60.3
Medium	2979	31.7	63.8	50.8	13.8	3.7	89.1	87.0	165 (50.8)	74.0	31.3	55.9	63.9
Large	7970	56.3	64.9	50.2	16.4	7.4	88.3	85.4	418 (44.7)	4.7	33.9	50.3	56.0
Southeast	38971	44.8	64.5	57.1	20.2	7.3	84.6	80.1	2,324 (38.6)	53.9	49.7	39.1	73.4
Small	12243	49.4	64.5	62.0	22.3	5.3	86.0	81.0	735 (43.0)	98.4	49.3	40.9	72.0
Medium	8880	40.3	64.1	56.7	21.5	7.0	84.8	81.4	546 (40.4)	58.2	45.7	41.5	74.1
Large	17848	44.4	64.7	54.1	18.0	8.8	83.4	78.7	1,043 (35.2)	21.1	51.9	36.7	74.1
South	24540	83.2	64.5	58.5	86.7	5.9	85.2	84.3	1,350 (37.3)	61.0	49.7	34.0	73.5
Small	12274	83.1	64.4	61.6	86.4	3.6	85.5	84.4	646 (36.3)	95.2	52.9	34.7	76.2
Medium	7257	82.9	64.2	57.2	87.5	5.9	85.0	83.6	418 (38.4)	41.0	45.1	37.1	71.3
Large	5009	84.1	65.0	52.9	86.3	11.3	85.0	84.7	286 (38.0)	6.3	48.2	27.9	70.3
Central-West	3740	25.7	63.2	56.5	22.2	4.4	87.0	83.3	226 (46.5)	53.3	50.4	33.9	69.5
Small	1475	26.8	62.7	60.1	22.0	2.8	88.3	82.2	74 (42.7)	100.0	50.9	36.0	69.3
Medium	777	25.4	63.0	57.3	22.3	5.5	86.4	80.6	52 (49.1)	55.5	44.1	31.5	76.1
Large	1488	24.8	63.6	52.6	22.4	5.4	86.1	85.8	100 (48.3)	5.8	53.2	33.1	66.3

**Table 3:** Mean and standard deviation of the bivariate analysis for the outcomes: absence of lung cancer cases in the municipality recorded in the Hospital Cancer Registry (RHC) relative to deaths recorded in the Mortality Information System (SIM) (Model 1), and lung cancer registration rate above the 3rd quartile (67.8) in the RHC (Model 2). Brazil, 2013-2019

Indicators	Model 1			Model 2		
	Total	No	Yes	Total	No	Yes
	Mean (Standard deviation)	Mean (Standard deviation)	Mean (Standard deviation)	Mean (Standard deviation)	Mean (Standard deviation)	Mean (Standard deviation)
Gross Domestic Product per capita (p-value<5%)	24626.4 ±25696.2	25336.2 ±26034.3	19658.1 ±22592.7	25336.2 ±26034.3	23363.3 ±27470.0	31258.0 ±20021.4
Education level (p-value<5%)	49 ±10.4	49.6 ±10.5	44.3 ±8.4	49.6 ±10.5	48.5 ±10.8	52.8 ±8.6
Life expectancy at birth (p-value< 5%)	73.1 ±2.7	73.3 ±2.7	72.1 ±2.5	73.3 ±2.6	72.7 ±2.6	74.8 ±1.9
Human Development Index	0.7 ±0.1	0.7 ±0.1	0.6 ±0.1	0.7 ±0.1	0.6 ±0.0	0.7 ±0.0
Supplementary health coverage (p-value<5%)	8.5 ±10.3	9.2 ±10.6	3.6 ±5.9	9.2 ±10.6	8.3 ±10.5	11.5 ±10.5
Physicians per 1,000 inhabitants (p-value<5%)	0.8 ±0.8	0.8 ±0.9	0.6 ±0.4	0.8 ±0.9	0.7 ±0.7	1.0 ±1.1
Rate of accredited health facilities per 100,000 inhabitants (p-value<5%)	- ±0.2	- ±0.2	-	- ±0.2	- ±0.1	- ±0.2
Urbanization (p-value<5%)	64 ±22	65.3 ±22.1	55.1 ±19.5	65.3 ±22.1	65.3 ±21.8	65.0 ±22.8
Primary health care coverage	92.1 ±16.9	91.6 ±17.2	96.1 ±13.8	91.6 ±17.2	0.7 ±0.4	0.8 ±0.4
Deaths outside SUS	3.6 ±9.1	3.7 ±8.7	2.7 ±11.4	3.7 ±8.7	3.7 ±8.7	3.6 ±8.5
Mean age (SIM) (p-value<5%)	68.2 ±5.8	68.1 ±5.3	68.7 ±8.6	-	-	-
Males (SIM) (p-value<5%)	62.1 ±19.2	62.4 ±17	59.9 ±30.3	-	-	-
Patients with higher education (SIM) (p-value<5%)	2.6 ±5.8	2.7 ±5.6	1.7 ±6.8	-	-	-
Mean age (RHC) (p-valor<5%)	-	-	-	64.0 ±6.7	63.7 ±7.1	64.8 ±4.8
Men (RHC) (p-valor<5%)	-	-	-	60.5 ±29.1	59.7 ±31.4	63.0 ±20.7
Patients with higher education (RHC) (p-valor<5%)	-	-	-	3.0 ±9.1	2.8 ±9.6	3.5 ±7.1
White patients (RHC) (p-valor<5%)	-	-	-	33.7 ±38.7	26.0 ±35.1	56.6 ±39.6
Advanced staging (p-value<5%)	-	-	-	86.9 ±19.6	87.4 ±20.9	85.4 ±14.5
Commuting	-	-	-	97.5 ±14.0	97.9 ±12.7	96.4 ±17.2
Timely care	-	-	-	15.9 ±21.1	15.9 ±22.7	15.7 ±15.1
Without diagnosis and treatment (p-value<5%)	-	-	-	44.9 ±33.3	42.2 ±34.2	52.7 ±29.3
With diagnosis but without treatment (p-value<5%)	-	-	-	44.4 ±32.3	47.4 ±33.6	36.8 ±26.7
Available without treatment (p-value<5%)	-	-	-	70.4 ±28.6	69.3 ±30.6	73.5 ±21.2

**Table 4:** Odds ratio (OR), adjusted odds ratio, and confidence intervals (95%) of the multivariate logistic regression models for the outcomes: absence of lung cancer cases in the municipality recorded in the Hospital Cancer Registry (RHC) relative to deaths recorded in the Mortality Information System (SIM) (Model 1), and lung cancer registration rate above the 3rd quartile in the RHC (67.8) (Model 2). 2013-2019

Indicators	Model 1 Absence of lung cancer cases in the municipal RHC				Model 2 Lung cancer registration rate above the 3rd quartile (67.8)			
	No. of municipalities in the analysis category	n (%)	OR (IC95%)	Adjusted OR (95%IC)	No. of municipalities in the analysis category	n (%)	OR (IC95%)	Adjusted OR (95%IC)
North region (reference category for Model 1)	42	86 (20.4)	-	-	-	-	-	-
North, Northeast, and Central-West regions (reference category for Model 2)	-	-	-	-	2,621	122 (5.0)	-	-
Northeast	1,751	265 (15.1)	0.69 (0.53; 0.91)	0.51 (0.38; 0.58)	-	-	-	-
Southeast	1,650	122 (7.3)	0.31 (0.23; 0.42)	0.59 (0.42; 0.82)	1,650	446 (27.0)	7.59 (6.14; 9.38)	4.66 (3.63; 5.99)
South	1,185	70 (5.9)	0.24 (0.17; 0.34)	0.47 (0.31; 0.72)	1,185	625 (52.7)	22.86 (18.45; 28.33)	9.08 (6.77; 12.17)
Central-West	450	139 (30.8)	1.74 (1.27; 2.37)	3.91 (2.67; 5.71)	-	-	-	-
Gross domestic product per capita^a^	3,546	542 (15.2)	2.28 (1.88; 2.77)	1.32 (1.01; 1.72)	-	-	-	-
Human Development Index	2,385	432 (18.1)	2.5 (2.11; 2.95)	1.66 (1.24; 2.22)	1,953	176 (9.0)	0.18 (0.15; 0.21)	0.6 (0.47; 0.76)
Supplementary health coverage	3,643	598 (16.4)	4.04 (3.19; 5.12)	1.58 (1.17; 2.13)	3,045	630 (20.7)	0.54 (0.47; 0.62)	1.27 (1.06; 1.53)
Physicians per 1,000 inhabitants	3,658	553 (15.1)	2.3 (1.89; 2.82)	1.02 (0.81; 1.28)	-	-	-	-
Mean age (SIM)	2,771	376 (13.5)	1.22 (1.04; 1.43)	1.17 (0.99; 1.39)	-	-	-	-
Percentage of lung cancer deaths in patients with higher education	4,002	626 (15.6)	4.63 (3.50; 6.13)	2.3 (1.71; 3.11)	-	-	-	-
Urbanization	2,641	450 (17)	2.29 (1.93; 2.71)	1.44 (1.17; 1.77)	-	-	-	-
Primary health care coverage	4,168	608 (14.5%)	2.8 (2.18; 3.60)	1.88 (1.43; 2.48)	-	-	-	-
Deaths outside SUS	4,255	627 (14.7%)	3.6 (2.71; 4.78)	2.01 (1.47; 2.74)	-	-	-	-
Small-sized	-	-	-	-	5,132	1,143 (22.3)	1.57 (1.11; 2.14)	7.05 (4.02; 12.35)
Percentage of patients with higher education (RHC)	-	-	-	-	962	348 (36.2)	1.99 (1.71; 2.32)	1.59 (1.31; 1.93)
Percentage of White patients with lung cancer (RHC)	-	-	-	-	1,821	778 (42.7%)	4.56 (3.97; 5.24)	1.75 (1.44; 2.13)
Advanced staging (RHC)	-	-	-	-	1,769	577 (32.6%)	1.88 (1.64; 2.14)	1.75 (1.49; 2.05)
Commuting (RHC)	-	-	-	-	169	58 (34.5%)	1.6 (1.15; 2.21)	4.15 (2.25; 7.65)
Timely care (RHC)	-	-	-	-	1,951	544 (27.9%)	1.3 (1.13; 1.48)	1.29 (1.11; 1.51)
Diagnosis without treatment (RHC)	-	-	-	-	2,409	759 (31.5%)	2.05 (1.79; 2.34)	1.8 (1.54; 2.10)
Mean age (RHC)	-	-	-	-	2,434	717 (29.5%)	1.64 (1.43; 1.87)	1.75 (1.50; 2.04)

## Discussion 

In Brazil, the highest lung cancer mortality rates are recorded in the South region, reaching up to three times those observed in the North. Furthermore, notable discrepancies are observed in the sociodemographic indicators of the North and Northeast regions compared to other regions, stratified by population size. It is also noteworthy that half of the patients diagnosed at early stages were unable to undergo the recommended treatment, and the vast majority were diagnosed at late stages. Additionally, localities with better infrastructure were more likely to have higher lung cancer registration rates, access to treatment, and greater "RHC/SIM coverage." In comparison, areas with poorer infrastructure were more likely not to register cases, despite recording deaths from lung cancer.

This study stands out for its innovation, as it is the first to analyze RHC/SIM coverage and its associated factors, as well as to contribute to the development of methodologies for data correction in the aforementioned databases. Regarding the study's limitations, the use of secondary data is noted, whose inferences are restricted to the variables available in the databases, which may exhibit incompleteness and inconsistencies. In this study, these issues were minimized by applying the corrections outlined in the methodology. Also, underreporting and differences in data collection across municipalities may occur. Finally, registration rates were calculated using the RHC, since there are no population-based databases with sufficient coverage and scope to calculate national incidence rates. Although the databases include only the public sector, the SUS (Unified Health System) has broad population coverage, particularly for cancer diagnosis and treatment. Therefore, the absence of data from the private sector does not significantly compromise the conclusions presented here ^12^. 

The findings of this study align with those reported in the literature, where RHC coverage outcomes relative to SIM and RHC registration rates exhibit the same uneven distribution pattern in access to health services, considering regional development and municipal population size across the country ^13^^,^^14^.

Access to treatment for individuals residing in small-sized municipalities in the South and Southeast regions was favored compared to those in large-sized municipalities, based on the finding of a significant difference between the crude OR and the adjusted OR for the variables "country region" and "population size" after modeling.

Data from the Brazilian National Health Survey (*Pesquisa Nacional em Saúde* - PNS) confirmed these findings ^15^, as they show that residents of the South and Southeast regions have greater access when compared to those from other regions, as well as individuals with higher levels of education. Other studies have pointed to significant differences in the availability of health services across Brazil, such as higher proportions of medical consultations in the South and Southeast, in addition to higher Human Development Index values ^13^. Meanwhile, the North and Northeast regions have lower sociodemographic indicators than those found in the other regions of the country ^13^^,^^14^.

A negative correlation was observed between lung cancer case registration rates and commuting time between the place of residence and the treatment center. It is hypothesized that municipalities with greater diagnostic and treatment capacity are those with better infrastructure for case registration. Furthermore, it was observed that, regarding geographical distribution, there is significant variability in the availability of accredited cancer treatment services, with the North region exhibiting the lowest installed capacity. Consequently, the distances traveled by patients living in rural areas, given Brazil's territorial dimensions, significantly compromise access to services ^6^. 

The findings of this study align with others in the literature, where socioeconomic factors and healthcare service availability determine inequalities in the distribution of lung cancer mortality rates. On the other hand, cancer stage at diagnosis is more closely associated with individual socioeconomic factors and tumor characteristics ^16^.

It is noteworthy that municipalities with a percentage of White individuals and higher education attainment below the average had higher odds of exhibiting a recorded rate above the 3rd quartile. Studies have shown that Black individuals face higher rates of lung cancer and different outcomes. The persistence of systemic and structural racism significantly contributes to these inequalities, which are associated with multiple factors that affect racial and ethnic groups ^17^. Higher education levels are associated with reduced smoking prevalence and greater success in quitting, which decreases the likelihood of lung cancer development ^4^.

In the United States, cancer patients from rural areas and those with lower socioeconomic status face greater barriers to accessing treatment. Furthermore, these localities present higher cancer rates associated with modifiable risk factors, such as smoking ^18^. 

In Brazil, the organization of healthcare services is a challenge, particularly in oncological care, as efficient coordination and collaboration among all levels of care is essential, along with a well-defined and regulated referral network for users. A study found that only 63% of the 27 Brazilian states had implemented their State Oncology Care Plans, and among these, most were not in compliance with established guidelines. Besides, states in the South and Southeast regions showed greater progress in developing and implementing these plans compared to those in the North and Northeast ^19^.

A promising alternative to facilitate patient access in the United States has been the use of satellite chemotherapy clinics, aimed at reducing the distance traveled to obtain treatment ^18^.

The proportion of patients who began treatment within the legal timeframe (60 days) ^10^ was 80%. Nonetheless, more than 85% of individuals were diagnosed at advanced stages ^20^. From the individual's perspective, this translates to worse survival outcomes, as the one-year survival rate is 63.5% when diagnosed at early stages, compared to 26.0% when diagnosed at advanced stages ^20^. For the health system, such a high rate of late diagnoses indicates significant delays and inefficiencies in the diagnostic process ^21^.

Factors associated with late diagnosis include disease pathophysiology, characterized by nonspecific symptoms that only manifest at advanced stages ^3^; shortcomings in medical training, which may lead to difficulties in interpreting radiological findings ^22^; the limited capacity and financial and human resources of the health system, which can delay diagnostic testing ^4^^,^^20^; and individual characteristics, such as perceptions and beliefs, stigma related both to smoking and to cancer, and fear of diagnosis, which may lead to delays in seeking care ^23^. In addition, in the Brazilian context, there is no national lung cancer screening policy, despite international recommendations ^7^.

In this study, the South region had the highest lung cancer rates in the country. Possible explanations for this result include longer life expectancy, lifestyle habits, particularly smoking, as well as better service structure and diagnostic capacity. Consequently, a greater number of diagnoses are observed, which may lead to increased registrations in official databases ^24^.

Municipalities where primary health care coverage is below the national average have higher odds of having recorded cases above the 3rd quartile. This inverse association can be explained by the fact that oncology services are concentrated in large urban centers, which generally have low primary health care coverage ^25^.

Despite the alarming global incidence and mortality figures for lung cancer ^1^, a decline in cancer cases and deaths has been observed for several types of cancer in regions with higher Human Development Index and better access to health services ^14^. Simultaneously, there is an increase in cases in regions with worse sociodemographic indicators, particularly for cancers associated with poverty, such as stomach, cervical, penile, and oral cavity cancers ^26^. This trend may reflect the impact of both socioeconomic inequalities and disparities in access to health services ^14^.

A significant proportion of individuals diagnosed at early stages did not undergo the recommended treatments of surgery and/or radiotherapy. In the North region, this proportion reached more than half of the cases. It is well known that Brazil has insufficient radiotherapy equipment and workforce ^27^. In terms of access to surgical treatment, it is a priority to adopt strategies that facilitate patient access to surgical procedures, improve professional training, enhance the safety of these procedures, and integrate surgical centers with units that perform other treatment modalities, such as chemotherapy and radiotherapy, thereby promoting comprehensive oncological care ^28^.

Probable factors affecting surgical access include socioeconomic disparities, comorbidities, advanced age, and the geographical distribution of healthcare services ^3^. With respect to human resources, it is noteworthy that thoracic surgeons are concentrated in the central-south regions of the country and in large cities ^29^. Consequently, even if the absolute number were adequate, their distribution would still be disproportionate, as medium-sized municipalities and densely populated regions in the Central, North, and Northeast of Brazil lack adequate coverage ^3^. This situation is concerning, as longer time intervals for performing surgical procedures as the initial treatment are directly associated with decreased overall survival in lung cancer ^30^.

Considering the findings on factors associated with case registration access, treatment characteristics, and lung cancer mortality, it is concluded that both service infrastructure and socioeconomic indicators were relevant for high lung cancer registration rates, access to treatment, and greater case coverage relative to deaths. Conversely, areas with poorer infrastructure in terms of high-complexity services were more likely not to register cases, despite recording deaths from lung cancer. 

Therefore, the results reflect the pattern of inequalities found in the country, the reduction of which is challenging. Since services with higher technological complexity are concentrated in certain regions, regulatory and contractual processes are crucial to ensure comprehensive access to cancer treatment. In addition to aspects related to the existence of infrastructure, it is necessary to create and implement strategies for training professionals in oncology and disseminating best practices in cancer care, enabling the expansion of timely access to diagnosis and treatment.

Given the presented scenario, it is suggested that future studies address the linkage of available data, analysis of patient therapeutic itineraries, and the impact of adopting screening strategies, including eligibility criteria.

## Data Availability

The database used in the research is available at: https://data.scielo.org/dataset.xhtml?persistentId=doi:10.48331/scielodata.8HSKF2.

## References

[1] 1. Bray F, Laversanne M, Sung H, Ferlay J, Siegel RL, Soerjomataram I, et al. Global cancer statistics 2022: GLOBOCAN estimates of incidence and mortality worldwide for 36 cancers in 185 countries. CA: A Cancer Journal for Clinicians. 2024;74(3):229-63. 10.3322/caac.21834 38572751

[2] 2. Instituto Nacional de Câncer José Alencar Gomes da Silva. Estimativa | 2023: Incidência de Câncer no Brasil [Internet]. Rio de Janeiro - RJ: INCA; 2022 [cited 2023 Jul 17]. Available from: Available from: https://www.inca.gov.br/sites/ufu.sti.inca.local/files/media/document/estimativa-2023.pdf .

[3] 3. Araujo LH, Baldotto C, Castro Jr G de, Katz A, Ferreira CG, Mathias C, et al. Lung cancer in Brazil. J bras pneumol. 2018 Feb;44(1):55-64. 10.1590/S1806-37562017000000135 PMC6104542 29538545

[4] 4. Campos MR, Rodrigues JM, Marques AP, Faria LV, Valerio TS, Silva MJS da, et al. Tabagismo, mortalidade, acesso ao diagnóstico e tratamento de câncer de pulmão no Brasil. Rev Saúde Pública. 2024 May 13;58:18. 10.11606/s1518-8787.2024058005704 PMC11090611 38747866

[5] 5. Kocher F, Hilbe W, Seeber A, Pircher A, Schmid T, Greil R, et al. Longitudinal analysis of 2293 NSCLC patients: A comprehensive study from the TYROL registry. Lung Cancer. 2015 Feb;87(2):193-200. 10.1016/j.lungcan.2014.12.006 25564398

[6] 6. Silva MJS da, O'Dwyer G, Osorio-de-Castro CGS. Cancer care in Brazil: structure and geographical distribution. BMC Cancer. 2019 Oct 23;19(1):987. 10.1186/s12885-019-6190-3 PMC6806503 31647005

[7] 7. Pereira LFF, Santos RS dos, Bonomi DO, Franceschini J, Santoro IL, Miotto A, et al. Recomendações da Sociedade Brasileira de Cirurgia Torácica, Sociedade Brasileira de Pneumologia e Tisiologia e Colégio Brasileiro de Radiologia e Diagnóstico por Imagem para o rastreamento do câncer de pulmão no Brasil. J bras pneumol. 2024 Mar 22;50:e20230233.

[8] 8. Skyrud KD, Bray F, Eriksen MT, Nilssen Y, Møller B. Regional variations in cancer survival: Impact of tumour stage, socioeconomic status, comorbidity and type of treatment in Norway. Int J Cancer. 2016 May 1;138(9):2190-200. 10.1002/ijc.29967 26679150

[9] 9. Tweed EJ, Allardice GM, McLoone P, Morrison DS. Socioeconomic inequalities in the incidence of four common cancers: a population-based registry study. Public Health. 2018 Jan;154:1-10. 10.1016/j.puhe.2017.10.005 PMC5764071 29128730

[10] 10. Brasil. Presidência da República. Diário Oficial da União. Sect. 1, Lei no 12732, de 22 de novembro de 2012 Nov 23, 2012 p. 1.

[11] 11. Rawal V, Kumar M, Verma A, Pais J. COVID-19 Lockdown: Impact on Agriculture and Rural Economy. Social Scientist. 2020;48(3/6 (562-565)):67-82.

[12] 12. Hetzel GM, Schwengber WK, Mondadori DC, Araujo LFL, Saueressig MG. Cirurgia de câncer de pulmão no Brasil durante a pandemia de COVID-19: Quantas ficaram para trás? J bras pneumol. 2022 Jul 25;48:e20220038. 10.36416/1806-3756/e20220038 PMC9496135 35894389

[13] 13. Stopa SR, Malta DC, Monteiro CN, Szwarcwald CL, Goldbaum M, Cesar CLG. Acesso e uso de serviços de saúde pela população brasileira, Pesquisa Nacional de Saúde 2013. Rev Saúde Pública. 2017 Jun 1;51:3s. 10.1590/S1518-8787.2017051000074 PMC5676359 28591351

[14] 14. Silva GA e, Jardim BC, Ferreira V de M, Junger WL, Girianelli VR. Mortalidade por câncer nas capitais e no interior do Brasil: uma análise de quatro décadas. Rev Saúde Pública. 2020 Dec 4;54:126. 10.11606/s1518-8787.2020054002255 PMC7688260 33295593

[15] 15. Viacava F, Bellido JG. Condições de saúde, acesso a serviços e fontes de pagamento, segundo inquéritos domiciliares. Ciênc saúde coletiva. 2016 Feb;21:351-70. 10.1590/1413-81232015212.19422015 26910144

[16] 16. Lima KYN de. Desigualdades na mortalidade e no estádio ao diagnóstico do câncer de pulmão no Brasil. 2021;146-146.

[17] 17. Brock BA, Mir H, Flenaugh EL, Oprea-Ilies G, Singh R, Singh S. Social and Biological Determinants in Lung Cancer Disparity. Cancers (Basel). 2024 Jan 31;16(3):612. 10.3390/cancers16030612 PMC10854636 38339362

[18] 18. Diaz A, Pawlik TM. Geographic Disparities in Oncologic Treatment and Outcomes: The Urban-Rural Divide. Ann Surg Oncol. 2021 Dec 1;28(13):8011-3. 10.1245/s10434-021-10653-3 34423403

[19] 19. Silva FA da, Trindade ES, Santiago BG, Afonso GV, Santos HLPC dos, Prado NM de BL. Políticas Públicas de Saúde para o Enfrentamento do Câncer no Brasil: Análise dos Planos Estaduais de Atenção Oncológica. Rev Bras Cancerol. 2025 Jan 13;70:e144454.

[20] 20. Valerio TS, Emmerick ICM, Sobreira-da-Silva MJ. Factors associated with late-stage diagnosis and overall survival for lung cancer: An analysis of patients treated in a Brazilian hospital and a US-hospital from 2009 to 2019. Cancer Epidemiol. 2023 Oct;86:102443. 10.1016/j.canep.2023.102443 37611485

[21] 21. Barros JA, Valladares G, Faria AR, Fugita EM, Ruiz AP, Vianna AGD, et al. Early diagnosis of lung cancer: the great challenge. Epidemiological variables, clinical variables, staging and treatment. J bras pneumol. 2006 Jun;32:221-7. 17273611

[22] 22. Ellis PM, Vandermeer R. Delays in the diagnosis of lung cancer. J Thorac Dis. 2011 Sep;3(3):183-8. 10.3978/j.issn.2072-1439.2011.01.01 PMC3256519 22263086

[23] 23. Albano D, Bilfinger T, Feraca M, Kuperberg S, Nemesure B. A Multidisciplinary Lung Cancer Program: Does It Reduce Delay Between Diagnosis and Treatment? Lung. 2020 Dec 1;198(6):967-72. 10.1007/s00408-020-00404-8 33159560

[24] 24. Panis C, Kawasaki ACB, Pascotto CR, Justina EYD, Vicentini GE, Lucio LC, et al. Revisão crítica da mortalidade por câncer usando registros hospitalares e anos potenciais de vida perdidos. einstein (São Paulo). 2018 Apr 23;16:eAO4018.

[25] 25. Macinko J, Mendonça CS. Estratégia Saúde da Família, um forte modelo de Atenção Primária à Saúde que traz resultados. Saúde debate. 2018 Sep;42:18-37.

[26] 26. Freitas LA de, Zanetti AC, Ramalho NS. O câncer e suas relações com a escolaridade e a pobreza. STUDIES IN HEALTH SCIENCES. 2024 Jul 25;5(3):e6075-e6075.

[27] 27. De Araújo LP, De Sá NM, Atty AT de M. Necessidades Atuais de Radioterapia no SUS e Estimativas para o Ano de 2030. Rev BrasileiraDeCancerologia. 2016;62(1):35-42.

[28] 28. Hoekstra HJ, Wobbes T, Heineman E, Haryono S, Aryandono T, Balch CM. Fighting Global Disparities in Cancer Care: A Surgical Oncology View. Ann Surg Oncol. 2016 Jul;23(7):2131-6. 10.1245/s10434-016-5194-3 PMC4889619 27038459

[29] 29. Tedde ML, Petrere O Jr, Pinto Filho DR, Pereira STLF, Monteiro R, Sassaki AM, et al. General thoracic surgery workforce: training, migration and practice profile in Brazil. European Journal of Cardio-Thoracic Surgery. 2015 Jan 1;47(1):e19-24. 10.1093/ejcts/ezu411 25391387

[30] 30. Johnson BA, Waddimba AC, Ogola GO, Fleshman JW, Preskitt JT. A systematic review and meta-analysis of surgery delays and survival in breast, lung and colon cancers: Implication for surgical triage during the COVID-19 pandemic. The American Journal of Surgery. 2021 Aug 1;222(2):311-8. 10.1016/j.amjsurg.2020.12.015 PMC7834494 33317814

