# Supplementary material for: Disparities in lung cancer case registration, treatment, and mortality: descriptive study, Brazil, 2013-2019
Source: Epidemiol Serv Saude. 2026 Apr 12;35:e20240909. doi: 10.1590/S2237-96222026v35e20240909.en (PMC13686316; doi:10.1590/S2237-96222026v35e20240909.en)
Supplement: Translation

# Translation: Disparidades no registro, no tratamento e na mortalidade por câncer de pulmão: estudo descritivo, Brasil, 2013-2019

**DOI:** 10.1590/S2237-96222026v35e20240909.pt

**Authors:** Lara Vinhal Faria, Isabel Martins Emmerick, Monica Rodrigues Campos

**Affiliations:** 1 Fundação Oswaldo Cruz, Escola Nacional de Saúde Pública Sérgio Arouca, Rio de Janeiro, RJ, Brasil Fundação Oswaldo Cruz Escola Nacional de Saúde Pública Sérgio Arouca Rio de Janeiro RJ Brasil; 2 Division of Thoracic Surgery, Department of Surgery, UMass Chan Medical School, Worcester, MA, Estados Unidos Division of Thoracic Surgery Department of Surgery UMass Chan Medical School Worcester MA Estados Unidos

**Keywords:** Neoplasias pulmonares, Registros de Mortalidade, Sistemas de Iinformação em Saúde, Disparidades Socioeconômicas em Saúde, Estudos Retrospectivos.

## Abstract

**Objetivo:**

Analisar fatores associados ao acesso do registro de casos, as características do tratamento e a mortalidade por câncer de pulmão de 2013-2019 no Brasil.

**Métodos:**

Estudo retrospectivo utilizando o Sistema de Informação de Mortalidade (SIM) e Registros Hospitalares de Câncer (RHC). Desfechos analisados: (Modelo-1) "ausência de registro de caso de câncer de pulmão" no RHC e (Modelo-2) "taxa de casos registrados acima do 3º quartil". Realizou-se análises descritivas e bivariadas, para comparar os grupos, e regressão logística multivariada, identificando-se, no nível municipal, fatores associados aos desfechos, obtendo-se a razão de chance ajustada (*odds ratio*, OR) e o intervalo de confiança de 95% (IC95%).

**Resultados:**

Analisou-se 206.703 óbitos e 89.265 casos (43% de cobertura). Cerca de 85,7% dos pacientes foram diagnosticados tardiamente, e metade dos casos com estadiamento inicial não recebeu tratamento adequado. No Modelo-1, a não cobertura do RHC foi associada a municípios do Centro-Oeste em comparação ao Norte [OR 3,9; IC95% 2,7; 5,7] e ao Produto Interno Bruto abaixo da média nacional [OR 1,3; IC95% 1,1; 1,7]. No Modelo-2, a maior chance de alta taxa de registro ocorreu em municípios do Sul [OR 9,1; IC95% 6,8; 12,2], de pequeno porte [OR 7,1; IC95% 4,0; 12,3] e com deslocamento para tratamento abaixo da média nacional [OR 4,2; IC95% 2,3; 7,7].

**Conclusão:**

Localidades com melhor infraestrutura apresentaram maior probabilidade de registrar casos e ter maior cobertura de registros. Já áreas com menor infraestrutura apresentaram maior probabilidade de não registrar casos, apesar de apresentarem óbitos por câncer de pulmão.

**Ethical aspects**

Esta pesquisa utilizou bancos de dados de domínio público e anonimizados.

## Introdução 

O câncer de pulmão, segundo estimativas mundiais para 2022, é o mais letal, com uma estimativa de 2,2 milhões de novos casos por ano e 1,8 milhões de mortes ^1^. No Brasil, estima-se que, para cada ano do triênio 2023-2025, haverá 30 mil novos casos ^2^.

O tabagismo é um importante fator de risco para o desenvolvimento da doença. Aproximadamente 85% dos casos estão associados ao tabaco. Por esse motivo, o Brasil tem como principal política pública para a redução da mortalidade iniciativas contra o tabagismo ^3^. 

No geral, o diagnóstico ocorre em estágios avançados e as taxas de sobrevida são baixas. Aproximadamente 85% dos pacientes apresentam doença localmente avançada ou metastática (estágio III e IV, respectivamente) no diagnóstico ^4^. O câncer de pulmão é de sintomatologia inespecífica, sendo um desafio para o diagnóstico nos estágios iniciais ^5^. Essa questão evidencia problemas no acesso aos serviços de oncologia e revela a carência na quantidade e na qualidade dos mesmos ^6^.

Atualmente, há um movimento para a implementação do rastreamento para o câncer de pulmão no Brasil, pois essa seria uma estratégia viável para a sua detecção em estágios iniciais e, consequentemente, para a redução da mortalidade ocasionada por ele ^7^.

A distribuição de equipamentos de diagnóstico por imagem e de centros especializados para o tratamento é desigual no Brasil, tendo consequências nos resultados em saúde para indivíduos que residem em diferentes localidades ^8^. Há evidência na literatura mundial que características sociodemográficas e econômicas precárias relacionam-se com uma menor sobrevida e um pior prognóstico ^9^. No entanto, verifica-se uma lacuna no conhecimento quanto a isso no Brasil.

Assim, propõe-se analisar o acesso ao registro de casos, as características do tratamento e a mortalidade por câncer de pulmão no período de 2013 a 2019, investigando fatores associados.

## Métodos 

Delineamento do estudo

Estudo retrospectivo, utilizando fontes de dados secundárias de abrangência nacional, sendo elas: censos do Instituto Brasileiro de Geografia e Estatística (IBGE) , dados do Programa das Nações Unidas para o Desenvolvimento (PNUD), dados do Sistema de Informações sobre Mortalidade (SIM) e os Registros Hospitalares de Câncer (RHC). Realizou-se a triangulação desses dados por município, segundo código de identificação do IBGE, e os casos de câncer de pulmão do RHC foram selecionados pelo local de residência, via código C34 (da 10ª Revisão da Classificação Estatística Internacional de Doenças e Problemas Relacionados à Saúde - CID-10). Toda a análise foi estratificada segundo número de habitantes por macrorregião: pequeno porte (menos de 100 mil); médio porte (entre 100 mil e 400 mil); grande porte (mais de 400 mil).

Variáveis

1) Indicadores sociodemográficos, estimados no censo demográfico de 2010 - IBGE e PNUD: Produto Interno Bruto (PIB) per capita no ano de 2019; escolaridade (percentual de pessoas de 25 anos ou mais com até 8 anos de escolaridade); urbanização (percentual de população urbana por município); esperança de vida

Para a cobertura da saúde suplementar, utilizou-se os dados da Agência Nacional de Saúde Suplementar, que expressa a porcentagem da população beneficiária de planos privados de saúde, valores de junho de 2019.

A cobertura da atenção básica foi obtida do sistema e-Gestor AB e da cobertura da saúde suplementar, e o número de médicos por 1 mil habitantes do Sistema de Informação de Beneficiários.

2) Casos de câncer de pulmão registrados no RHC de 2013 a 2019 e indicadores clínicos: estadiamento avançado câncer de pulmão (percentual de indivíduos com estadiamento III e IV); perda de oportunidade (percentual de pacientes com estadiamento inicial que não fizeram cirurgia ou radioterapia); tempo adequado (percentual de indivíduos com tempo para o início do tratamento em até 60 dias após o diagnóstico) ^10^; deslocamento (percentual de pacientes que realizaram o tratamento fora do município de residência); idade; percentual de pessoas: brancas, com nível superior, com diagnóstico e sem tratamento, sem diagnóstico e sem tratamento e pacientes tratados. 

No cálculo do tempo adequado, utilizou-se o critério mais favorável possível para definir a data de primeiro contato com o serviço, a data da primeira consulta ou a data da realização da triagem, o que apresentasse o menor tempo, e excluiu-se desta análise os não elegíveis para tratamento, por estarem fora de possibilidade terapêutica.

3) Caracterização dos óbitos por câncer de pulmão, no SIM, de 2013 a 2019, com o cálculo da taxa de mortalidade por câncer de pulmão por 100 mil habitantes: média de idade; percentual de óbitos do sexo masculino; percentual de óbitos de nível superior.

Optou-se por utilizar o período de 2013 a 2019 devido à consistência e à qualidade das informações do RHC nesse período e para evitar o efeito da pandemia de covid-19, tanto nos serviços de saúde quanto no padrão de busca de cuidado ^11^.

As técnicas de correção tanto do SIM quanto do RHC são descritas em detalhes em artigo prévio ^4^.

Métodos estatísticos

Para identificação dos fatores associados à disparidade no registro de casos e de óbitos, utilizou-se como desfecho a "ausência de registro de caso de câncer de pulmão" no RHC e aplicou-se modelo de regressão logística multivariado denominado Modelo 1. Para caracterizar o acesso ao tratamento e aspectos de sua qualidade no RHC, após análise de sensibilidade, escolheu-se o desfecho "taxa de casos registrados de câncer de pulmão acima do 3º quartil", no período de 2013 a 2019, por população estimada para 2019, segundo municípios de residência, via modelo de regressão logística multivariado (Modelo 2).

Na análise descritiva e bivariada, caracterizou-se as variáveis de exposição a partir da sua média, o desvio padrão e o valor mínimo e o máximo, segundo as categorias investigadas em cada desfecho.

Para as variáveis de exposição contínuas, realizou-se teste-t de Student de diferença de média, no software SPSS v21 ao nível de 5% (p-valor<5%), seguidas de dicotomização das mesmas em valores "0" e "1". 

Definiu-se a categoria de referência em cada variável seguindo-se o padrão observado na categoria do desfecho. Assim, se o valor da variável era maior na categoria "1" do desfecho, então se atribuía o código "1" relativo ao valor "acima da média nacional". Então, todas as variáveis de exposição analisadas tiveram como categoria de referência valores acima da média nacional, exceto para: percentual de pacientes de câncer de pulmão brancos (RHC); média de idade (RHC); percentual de pacientes com ensino superior (RHC); média de idade (SIM); cobertura da atenção básica. Dessa forma, apresentaram-se as razões de chances brutas (OR bruto) e respectivos intervalos de confiança de 95% (IC95%) como um aumento da razão de chance quando comparado ao valor de referência.

No processo de construção dos modelos, buscou-se, em princípio, modelar as taxas de cobertura. Já em processos iterativos de modelagem de análises de sensibilidade, chegou-se aos dois modelos finais, ajustados por variáveis significativas estatisticamente, dentre aquelas pertencentes ao modelo teórico explicativo do acesso. Assim, as OR ajustadas foram obtidas a partir da realização das regressões logísticas, no Modelo 1, via método enter e, no Modelo-2, com seleção de variáveis via método stepwise backward.

Os bancos de dados foram tratados utilizando-se a linguagem de programação Python v.3.7. Utilizou-se SPSS v21 para as análises estatísticas.

**Figura 1:** Municípios sem casos registrados de óbito no Sistema de Informações em Mortalidade (SIM) (A); número de casos registrados nos Registros Hospitalares de Câncer (RHC) em relação ao número de óbitos por câncer de pulmão do SIM (B); taxa de registros de câncer de pulmão por 100 mil habitantes (RHC) (C); taxa de óbitos por câncer de pulmão por 100 mil habitantes do SIM (D). Brasil, 2013-2019

## Resultados 

No Brasil, após as correções, foram registrados 206.703 óbitos por câncer de pulmão no período de 2013 a 2019, e a taxa de mortalidade foi de 99,4 por 100 mil habitantes. A maior taxa de mortalidade foi registrada na região Sul (161,7 por 100 mil habitantes), chegando a ser três vezes maior do que a da região Norte (54,8 por 100 mil habitantes), a menor do país (Figure 1).

O percentual de correção no SIM foi de 0,03% na variável sexo, 2% para a idade, 2% nos códigos-lixo, 6% nas causas mal definidas e 4,7% nos subregistros. Ainda que pequenas, as correções no SIM foram importantes para melhor completude da informação, e a correção realizada no RHC para o estadiamento foi fundamental, evitando 32% de perda.

Quanto aos indicadores sociodemográficos por região e por porte populacional, vê-se acentuadas disparidades e presença de gradientes segundo o porte dentro de cada região. Destaca-se que o PIB per capita médio dos municípios de grande porte das regiões Norte e Nordeste se equiparam ao PIB per capita médio dos municípios de pequeno porte das demais regiões, configurando uma enorme disparidade de recursos financeiros, humanos e de infraestrutura. Além disso, em termos da distribuição dos indicadores de saúde, ressalta-se que a cobertura da Atenção Básica varia de 61,4% no grande porte até 93,7% no pequeno porte, enquanto que a cobertura da saúde suplementar se distribuiu de forma inversamente proporcional (Tabela 1).

No mesmo período, registraram-se 89.265 casos de câncer de pulmão no RHC, com mais de 80% dos pacientes começando o tratamento dentro de 60 dias após o diagnóstico, o que é majoritariamente tardio (85,7% de estadiamento avançado). Na região Norte, mais de 50% dos pacientes não realizaram cirurgia ou radioterapia como primeiro tratamento no estadiamento inicial (Tabela 2).

No período investigado, detectou-se cerca de 43% de cobertura de registros de câncer de pulmão no RHC em relação aos óbitos do SIM ("cobertura RHC/SIM"), evidenciando uma diferença significativa entre o número de casos registrados e os óbitos por câncer de pulmão.

O Centro-Oeste, com destaque para Goiás, apresentou maior número de municípios com registro de óbitos por câncer de pulmão, sem o registro de casos no RHC. Notou-se que os estados que têm maiores áreas com "cobertura RHC/SIM" abaixo de 25% são: Goiás, Amazonas e Acre. Quanto às taxas de registro no RHC e de mortalidade no SIM, observou-se mais municípios com altas taxas localizados nas regiões Sul e Sudeste (Figure 1).

Na análise bivariada, observou-se que municípios com "ausência de registro de casos" (desfecho do Modelo 1) apresentaram "piores" resultados sociodemográficos e de saúde. Já aqueles com taxas de registro de câncer de pulmão acima do 3º quartil (desfecho do Modelo 2) apresentaram indicadores sociodemográficos mais "altos" quando comparados aos demais, além de menor percentual de estadiamento avançado, de deslocamento e de pacientes com diagnóstico e sem tratamento (Tabela 3).

No Modelo 1, de regressão logística multivariada, os municípios da região Centro-Oeste, comparados aos da Norte, apresentaram mais chances de ter ausência de casos de câncer de pulmão no RHC, assim como aqueles com percentual de óbitos sem vínculo com o SUS (setor privado), percentual de pessoas com ensino superior (no SIM) com valores abaixo da média nacional, e àqueles onde a cobertura da atenção básica está acima da média nacional (Tabela 4).

No Modelo 2, as variáveis relacionadas com uma maior chance de apresentar o desfecho foram: ser da região Sul; ser de pequeno porte; ter um deslocamento abaixo da média nacional. Um dos maiores OR ajustada observado no Modelo 2 foi para a variável "deslocamento entre município para tratar", com 4,2 vezes mais chances de o município ter "alta" taxa de registro quando a porcentagem de deslocamento está abaixo da média nacional (Tabela 4). 

Indivíduos brancos, com maior escolaridade e mais velhos, além dos que moram em localidades com maior infraestrutura para diagnóstico e tratamento, apresentaram maiores chances de ter casos de câncer de pulmão registrados acima do 3º quartil (Tabela 4).

**Tabela 1:** Distribuição dos óbitos por câncer de pulmão e indicadores sociodemográficos por região e porte municipal. Brasil, 2013-2019

Porte municipal	Total de óbitos	Taxa de mortalidade/100 mil habitantes	Sexo masculino (%)	Ensino superior (%)	Média de idade	PIB per capita	Escolaridade (%)	Esperança de vida ao nascer	IDH^a^	Urbanização (%)	Médicos 1 mil habitantes	Cobertura atenção básica (%)	Cobertura saúde suplementar (%)	Óbito sem vínculo SUS (%)
Brasil	206.703	99,4	57,9	7,5	68,1	24.539,90	48,9	73,06	0,66	63,8	2,0	92,2	22,5	11,1
Pequeno	83.420	94,4	60,3	3,1	68,3	23.730,38	47,8	72,93	0,65	62,0	0,9	93,7	9,5	3,9
Médio	46.825	99,3	57,7	6,1	67,4	36.631,09	64,7	75,09	0,73	91,9	1,9	69,9	24,1	9,6
Grande	76.458	105,5	55,5	13,1	68,3	41.842,99	72,4	75,45	0,77	98,0	3,2	61,4	37,4	19,9
Região Norte	9.869	54,8	60,0	4,7	67,6	18.415,07	47,4	71,82	0,61	56,7	1,0	87,5	9,4	7,9
Pequeno	4.008	46,5	62,3	2,2	67,4	17.911,74	46,5	71,73	0,60	55,0	0,5	88,9	2,1	4,3
Médio	1.649	43,1	62,4	3,5	67,4	25.369,85	57,8	72,82	0,66	76,5	1,1	70,6	8,6	5,6
Grande	4.212	75,6	57,0	7,5	67,8	25.828,52	71,3	74,05	0,73	96,2	1,8	62,7	21,4	12,2
Região Nordeste	42.424	75,0	53,9	5,2	68,5	12.584,70	40,1	70,17	0,59	55,2	1,3	97,0	11,6	4,5
Pequeno	22.567	68,4	55,0	1,7	68,9	12.221,96	39,4	70,06	0,59	54,0	0,6	97,6	2,8	1,6
Médio	7.138	76,0	53,0	4,8	68,3	21.850,04	57,4	72,88	0,68	86,1	1,5	83,7	14,4	4,1
Grande	12.719	89,9	52,5	11,6	67,8	25.502,54	68,6	74,33	0,75	97,8	2,8	65,9	30,4	9,9
Região Sudeste	92.831	105,9	57,9	9,8	68,1	27.816,66	54,4	74,68	0,70	74,9	2,5	88,9	32,5	16,7
Pequeno	26.285	103,5	60,8	4,4	68,2	26.197,51	52,8	74,56	0,69	72,7	1,2	91,7	18,1	5,5
Médio	22.040	100,1	58,6	7,4	67,3	42.383,80	68,5	75,95	0,76	96,1	2,2	62,8	31,5	13,5
Grande	44.506	110,6	55,9	14,1	68,5	48.688,28	73,0	75,84	0,78	98,5	3,4	58,7	42,2	24,8
Região Sul	48.079	161,7	60,7	5,5	68,0	36.247,70	53,4	75,12	0,71	60,7	2,2	91,2	23,2	6,3
Pequeno	25.620	170,4	63,5	3,1	68,1	35.843,55	52,7	75,05	0,71	59,1	1,2	92,1	13,9	3,8
Médio	13.652	156,0	58,3	5,4	67,2	44.284,70	67,3	76,42	0,76	94,1	2,3	74,2	25,1	7,0
Grande	8.807	147,8	56,0	12,8	68,9	49.135,75	76,6	76,61	0,81	97,8	4,5	71,3	43,9	12,4
Região Centro-Oeste	13.500	84,1	59,3	8,0	67,4	34.780,18	52,9	74,35	0,69	71,9	2,0	92,9	20,1	13,4
Pequeno	4.940	78,3	62,8	2,6	67,6	34.640,97	52,2	74,30	0,69	70,6	0,9	94,5	10,6	6,7
Médio	2.346	73,9	57,8	4,3	67,3	35.365,75	63,1	75,15	0,73	94,6	1,5	67,6	18,0	6,9
Grande	6.214	94,6	57,0	13,7	67,4	44.888,69	72,8	75,65	0,78	98,5	3,3	52,2	30,4	21,2

a Índice de desenvolvimento humano.

**Tabela 2:** Distribuição dos casos de câncer de pulmão, taxa de casos registrados, indicadores clínicos, sociodemográficos por região e porte municipal. Brasil, 2013-2019

Porte municipal	Total de casos	Taxa de incidência por 100 mil habitantes	Média de idade	Pacientes homens (%)	Pacientes brancos (%)	Pacientes com ensino superior (%)	Estadiamento avançado (%)	Tempo adequado (%)	Perda de oportunidade n(%)	Deslocamento (%)	Sem diagnóstico e tratamento (%)	Com diagnóstico e sem tratamento (%)	Pacientes tratados (%)
Brasil	89.265	44,2	64,4	56,5	37,1	6,1	85,7	82,7	5.166 (40,5)	56,0	46,1	40,7	70,1
Pequeno	34.878	42,1	64,2	60,1	42,5	3,7	86,3	83,3	1.984 (41,6)	97,5	47,3	41,6	70,8
Médio	20.596	43,8	64,1	56,1	43,4	6,0	85,6	83,1	1.231 (41,6)	54,4	43,3	41,8	71,7
Grande	33.791	47,0	64,7	53,1	27,7	8,6	85,1	81,8	1.951 (38,7)	14,1	46,5	39,1	68,3
Região Norte	3.470	20,8	63,7	58,9	14,1	4,5	86,9	84,8	254 (55,9)	54,7	39,1	50,7	67,8
Pequeno	1.291	17,8	63,1	59,5	17,4	2,5	86,1	85,1	100 (55,9)	97,2	38,1	51,4	72,3
Médio	703	18,4	64,7	59,6	13,2	5,5	87,6	83,9	50 (57,5)	59,9	45,0	46,1	73,7
Grande	1.476	26,5	63,8	58,0	11,6	5,8	87,3	85,0	104 (55,3)	15,0	37,2	52,3	60,9
Região Nordeste	18.542	34,3	64,2	52,1	14,2	4,5	88,2	85,5	1.012 (46,3)	54,6	34,2	52,4	59,0
Pequeno	7.593	24,9	63,8	54,6	12,1	1,7	87,8	85,1	429 (46,4)	99,4	35,7	53,3	60,3
Médio	2.979	31,7	63,8	50,8	13,8	3,7	89,1	87,0	165 (50,8)	74,0	31,3	55,9	63,9
Grande	7.970	56,3	64,9	50,2	16,4	7,4	88,3	85,4	418 (44,7)	4,7	33,9	50,3	56,0
Região Sudeste	38.971	44,8	64,5	57,1	20,2	7,3	84,6	80,1	2.324 (38,6)	53,9	49,7	39,1	73,4
Pequeno	12.243	49,4	64,5	62,0	22,3	5,3	86,0	81,0	735 (43,0)	98,4	49,3	40,9	72,0
Médio	8.880	40,3	64,1	56,7	21,5	7,0	84,8	81,4	546 (40,4)	58,2	45,7	41,5	74,1
Grande	17.848	44,4	64,7	54,1	18,0	8,8	83,4	78,7	1.043 (35,2)	21,1	51,9	36,7	74,1
Região Sul	24.540	83,2	64,5	58,5	86,7	5,9	85,2	84,3	1.350 (37,3)	61,0	49,7	34,0	73,5
Pequeno	12.274	83,1	64,4	61,6	86,4	3,6	85,5	84,4	646 (36,3)	95,2	52,9	34,7	76,2
Médio	7.257	82,9	64,2	57,2	87,5	5,9	85,0	83,6	418 (38,4)	41,0	45,1	37,1	71,3
Grande	5.009	84,1	65,0	52,9	86,3	11,3	85,0	84,7	286 (38,0)	6,3	48,2	27,9	70,3
Centro-Oeste	3.740	25,7	63,2	56,5	22,2	4,4	87,0	83,3	226 (46,5)	53,3	50,4	33,9	69,5
Pequeno	1.475	26,8	62,7	60,1	22,0	2,8	88,3	82,2	74 (42,7)	100,0	50,9	36,0	69,3
Médio	777	25,4	63,0	57,3	22,3	5,5	86,4	80,6	52 (49.1)	55,5	44,1	31,5	76,1
Grande	1.488	24,8	63,6	52,6	22,4	5,4	86,1	85,8	100 (48,3)	5,8	53,2	33,1	66,3

**Tabela 3:** Média e desvio padrão da análise bivariada para os desfechos: ausência de casos de câncer de pulmão do município registrados no Registro Hospitalar de cancer (RHC) em relação aos óbitos registrados no Sistema de Informação de Mortalidade (SIM) (Modelo 1) e taxa de registro de câncer de pulmão acima do 3º quartil (67,8) no RHC (Modelo 2). Brasil, 2013-2019

Indicadores	Modelo 1			Modelo 2		
	Total	Não	Sim	Total	Não	Sim
	Média (Desvio padrão)	Média (Desvio padrão)	Média (Desvio padrão)	Média (Desvio padrão)	Média (Desvio padrão)	Média (Desvio padrão)
Produto Interno Bruno per capita (p-valor<5%)	24626,4 ±25696,2	25336,2 ±26034,3	19658,1 ±22592,7	25336,2 ±26034,3	23363,3 ±27470,0	31258,0 ±20021,4
Escolaridade (p-valor<5%)	49 ±10,4	49,6 ±10,5	44,3 ±8,4	49,6 ±10,5	48,5 ±10,8	52,8 ±8,6
Esperança de vida ao nascer (p-valor<5%)	73,1 ±2,7	73,3 ±2,7	72,1 ±2,5	73,3 ±2,6	72,7 ±2,6	74,8 ±1,9
Índice de Desenvolvimento Humano (p-valor<5%)	0,7 ±0,1	0,7 ±0,1	0,6 ±0,1	0,7 ±0,1	0,6 ±0,0	0,7 ±0,0
Cobertura da saúde suplementar (p-valor<5%)	8,5 ±10,3	9,2 ±10,6	3,6 ±5,9	9,2 ±10,6	8,3 ±10,5	11,5 ±10,5
Médicos por 1 mil habitantes (p-valor<5%)	0,8 ±0,8	0,8 ±0,9	0,6 ±0,4	0,8 ±0,9	0,7 ±0,7	1,0 ±1,1
Taxa de estabelecimentos de saúde habilitados por 100 mil habitantes (p-valor<5%)	- ±0,2	- ±0,2	-	- ±0,2	- ±0,1	- ±0,2
Urbanização (p-valor<5%)	64 ±22	65,3 ±22,1	55,1 ±19,5	65,3 ±22,1	65,3 ±21,8	65,0 ±22,8
Cobertura da atenção básica	92,1 ±16,9	91,6 ±17,2	96,1 ±13,8	91,6 ±17,2	0,7 ±0,4	0,8 ±0,4
Óbito sem vínculo com o SUS	3,6 ±9,1	3,7 ±8,7	2,7 ±11,4	3,7 ±8,7	3,7 ±8,7	3,6 ±8,5
Média de idade (SIM) (p-valor<5%)	68,2 ±5,8	68,1 ±5,3	68,7 ±8,6	-	-	-
Pessoas do sexo masculino (SIM) (p-valor<5%)	62,1 ±19,2	62,4 ±17	59,9 ±30,3	-	-	-
Pessoas com ensino superior (SIM) (p-valor<5%)	2,6 ±5,8	2,7 ±5,6	1,7 ±6,8	-	-	-
Média de idade (RHC) (p-valor<5%)	-	-	-	64,0 ±6,7	63,7 ±7,1	64,8 ±4,8
Homens (RHC) (p-valor<5%)	-	-	-	60,5 ±29,1	59,7 ±31,4	63,0 ±20,7
Pacientes com ensino superior (RHC) (p-valor<5%)	-	-	-	3,0 ±9,1	2,8 ±9,6	3,5 ±7,1
Pacientes brancos (RHC) (p-valor<5%)	-	-	-	33,7 ±38,7	26,0 ±35,1	56,6 ±39,6
Estadiamento avançado (p-valor<5%)	-	-	-	86,9 ±19,6	87,4 ±20,9	85,4 ±14,5
Deslocamento	-	-	-	97,5 ±14,0	97,9 ±12,7	96,4 ±17,2
Tempo adequado	-	-	-	15,9 ±21,1	15,9 ±22,7	15,7 ±15,1
Sem diagnóstico e sem tratamento (p-valor<5%)	-	-	-	44,9 ±33,3	42,2 ±34,2	52,7 ±29,3
Com diagnóstico e sem tratamento (p-valor<5%)	-	-	-	44,4 ±32,3	47,4 ±33,6	36,8 ±26,7
Disponível sem tratamento (p-valor<5%)	-	-	-	70,4 ±28,6	69,3 ±30,6	73,5 ±21,2

**Tabela 4:** *Odds ratio* (OR), *odds ratio* ajustada e intervalos de confiança (95%) dos modelos de regressão logística multivariada para os desfechos: ausência de casos de câncer de pulmão do município registrados no Registro Hospitalar de Câncer (RHC) em relação aos óbitos registrados no Sistema de Informação de Mortalidade (SIM) (Modelo 1) e taxa de registro de câncer de pulmão acima do 3º quartil no RHC (67,8) (Modelo 2). Brasil, 2013-2019

Indicadores	Modelo 1 Ausência de casos de câncer de pulmão no RHC do município				Modelo 2 Taxa de registro de câncer de pulmão acima do 3º quartil (67,8)			
	Nº de municípios categoria-análise	n (%)	OR (IC95%)	OR ajustada (IC95%)	Nº de municípios categoria-análise	n (%)	OR (IC95%)	OR ajustada (IC95%)
Região Norte (categoria-referência do modelo 1)	42	86 (20,4)	-	-	-	-	-	-
Região Norte, Nordeste e Centro-Oeste (categoria-referência do modelo 2)	-	-	-	-	2.621	122 (5,0)	-	-
Região Nordeste	1.751	265 (15,1)	0,69 (0,53; 0,91)	0,51 (0,38; 0,58)	-	-	-	-
Região Sudeste	1.650	122 (7,3)	0,31 (0,23; 0,42)	0,59 (0,42; 0,82)	1.650	446 (27,0)	7,59 (6,14; 9,38)	4,66 (3,63; 5,99)
Região Sul	1.185	70 (5,9)	0,24 (0,17; 0,34)	0,47 (0,31; 0,72)	1.185	625 (52,7)	22,86 (18,45; 28,33)	9,08 (6,77; 12,17)
Região Centro-Oeste	450	139 (30,8)	1,74 (1,27; 2,37)	3,91 (2,67; 5,71)	-	-	-	-
Produto Interno Bruno per capitaa	3.546	542 (15,2)	2,28 (1,88; 2,77)	1,32 (1,01; 1,72)	-	-	-	-
Índice de Desenvolvimento Humano	2.385	432 (18,1)	2,5 (2,11; 2,95)	1,66 (1,24; 2,22)	1.953	176 (9,0)	0,18 (0,15; 0,21)	0,6 (0,47; 0,76)
Cobertura da Saúde Suplementar	3.643	598 (16,4)	4,04 (3,19; 5,12)	1,58 (1,17; 2,13)	3.045	630 (20,7)	0,54 (0,47; 0,62)	1,27 (1,06; 1,53)
Médico por 1 mil habitantes	3.658	553 (15,1)	2,3 (1,89; 2,82)	1,02 (0,81; 1,28)	-	-	-	-
Média de idade (SIM)	2.771	376 (13,5)	1,22 (1,04; 1,43)	1,17 (0,99; 1,39)	-	-	-	-
Percentual de óbitos por câncer de pulmão em pacientes com ensino superior	4.002	626 (15,6)	4,63 (3,50; 6,13)	2,3 (1,71; 3,11)	-	-	-	-
Urbanização	2.641	450 (17)	2,29 (1,93; 2,71)	1,44 (1,17; 1,77)	-	-	-	-
Cobertura da atenção básica	4.168	608 (14,5%)	2,8 (2,18; 3,60)	1,88 (1,43; 2,48)	-	-	-	-
Óbito sem vínculo com o SUS	4.255	627 (14,7%)	3,6 (2,71; 4,78)	2,01 (1,47; 2,74)	-	-	-	-
Pequeno porte	-	-	-	-	5.132	1.143 (22,3)	1,57 (1,11; 2,14)	7,05 (4,02; 12,35)
Percentual de pacientes com ensino superior (RHC)	-	-	-	-	962	348 (36,2)	1,99 (1,71; 2,32)	1,59 (1,31; 1,93)
Percentual de pacientes de câncer de pulmão brancos (RHC)	-	-	-	-	1.821	778 (42,7%)	4,56 (3,97; 5,24)	1,75 (1,44; 2,13)
Estadiamento avançado (RHC)	-	-	-	-	1.769	577 (32,6%)	1,88 (1,64; 2,14)	1,75 (1,49; 2,05)
Deslocamento (RHC)	-	-	-	-	169	58 (34,5%)	1,6 (1,15; 2,21)	4,15 (2,25; 7,65)
Tempo adequado (RHC)	-	-	-	-	1.951	544 (27,9%)	1,3 (1,13; 1,48)	1,29 (1,11; 1,51)
Diagnóstico sem tratamento (RHC)	-	-	-	-	2.409	759 (31,5%)	2,05 (1,79; 2,34)	1,8 (1,54; 2,10)
Média de idade (RHC)	-	-	-	-	2.434	717 (29,5%)	1,64 (1,43; 1,87)	1,75 (1,50; 2,04)

## Discussão 

No Brasil, as maiores taxas de mortalidade estão registradas na região Sul, sendo até três vezes maiores que na Norte. Adicionalmente, nota-se que há grandes discrepâncias nos indicadores sociodemográficos das regiões Norte e Nordeste quando comparados às demais, segundo porte populacional. Destaca-se, ainda, que metade dos pacientes com estadiamento inicial não consegue realizar o tratamento preconizado, bem como a grande maioria deles são diagnosticados tardiamente. Ademais, localidades com melhor infraestrutura apresentaram maior probabilidade de ter altas taxas de registro de câncer de pulmão, acesso ao tratamento e maior "cobertura RHC/SIM", enquanto áreas com menor infraestrutura apresentaram maior probabilidade de não registrar casos, apesar de registrarem óbitos por câncer de pulmão.

Este estudo destaca-se por sua inovação, sendo o primeiro a analisar a cobertura do RHC/SIM e seus fatores associados, além de contribuir para o desenvolvimento de metodologias para correção de dados nos bancos supracitados. Quanto às limitações do estudo, aponta-se a utilização de dados secundários, cujas inferências ficam restritas às variáveis existentes nos bancos, que podem apresentar incompletudes e inconsistências, as quais, neste estudo, foram minimizadas pela aplicação das correções apontadas na metodologia. Além disso, pode ocorrer subnotificação e diferenças na coleta da dados entre os municípios. Por fim, calculou-se a taxa de registro via RHC, pois não há disponibilidade de bases de dados populacionais com cobertura e abrangência suficiente para cálculo da taxa de incidência nacional. Ainda que os bancos de dados incluam somente o setor público, o SUS possui alta cobertura populacional, especialmente para o diagnóstico e o tratamento de câncer, assim, a ausência do setor privado não causa prejuízo significativo às conclusões aqui apresentadas ^12^. 

O panorama observado neste estudo é similar ao descrito na literatura, no qual os desfechos de cobertura do RHC em relação ao SIM e a taxa de registro no RHC seguem o mesmo padrão díspar de distribuição no acesso aos serviços de saúde, considerando o desenvolvimento das regiões e o porte populacional no país ^13^^,^^14^.

O acesso ao tratamento por indivíduos que residem em municípios de pequeno porte nas regiões Sul e Sudeste foi favorecido em comparação àqueles que residem em municípios de grande porte, a partir da constatação de diferença significativa entre o OR bruto e o OR ajustada das variáveis "região do país" e "porte populacional" após a modelagem.

Os dados da Pesquisa Nacional em Saúde (PNS) confirmaram esses achados ^15^, pois mostram que residentes das regiões Sul e Sudeste têm maior acesso quando comparados aos de outras regiões, bem como pessoas com maior escolaridade. Outros estudos sinalizaram grandes diferenças na disponibilidade de serviços de saúde entre as regiões do Brasil, como maiores proporções de consultas médicas nas regiões Sul e Sudeste, além de maior IDH ^13^. Enquanto isso, observa-se que as regiões Norte e Nordeste apresentam seus indicadores sociodemográficos mais baixos do que os encontrados nas outras regiões do país ^13^^,^^14^.

O registro de casos de câncer de pulmão correlacionou-se inversamente com o tempo de deslocamento entre local de residência e de tratamento. Hipotetiza-se que os municípios com melhor capacidade resolutiva para diagnóstico e tratamento são aqueles que possuem uma melhor estrutura para o registro. Observou-se ainda que, em relação à distribuição geográfica, há uma importante variabilidade na oferta de serviços habilitados para tratamento do câncer, sendo a região Norte a que apresenta menor capacidade instalada. Logo, a distância percorrida por pacientes residentes em áreas rurais, dadas as dimensões do Brasil, comprometem o acesso aos serviços de forma significativa ^6^. 

Os achados deste estudo se alinham a outros da literatura, em que fatores socioeconômicos e de oferta de serviços de saúde determinam desigualdades na distribuição das taxas de mortalidade por câncer de pulmão. Por outro lado, o estágio do câncer no diagnóstico está mais associado aos fatores individuais socioeconômicos e do próprio do tumor ^16^.

Ressalta-se que municípios com percentual de pessoas brancas e de ensino superior abaixo da média tiveram maiores chances de apresentarem taxa registrada acima do 3º quartil. Estudos mostram que pessoas negras enfrentam maiores taxas de câncer de pulmão e desfechos diferentes. A persistência do racismo sistêmico e estrutural contribui significativamente para essas desigualdades, associadas a diversos fatores que afetam grupos raciais e étnicos ^17^. Indivíduos com maior nível de escolaridade apresentam menor consumo de tabaco e uma chance aumentada na cessação do fumo, diminuindo o risco de desenvolvimento de câncer de pulmão ^4^.

Nos Estados Unidos, pacientes oncológicos advindos de áreas rurais e aqueles de baixo nível socioeconômico têm o acesso ao tratamento dificultado. Adicionalmente, essas localidades têm maiores taxas de câncer associadas a fatores de risco modificáveis, como o tabagismo ^18^. 

No Brasil, a organização dos serviços de saúde é um desafio, especialmente no cuidado oncológico, pois a pactuação e a articulação eficiente entre todos os níveis de atenção é essencial, assim como uma rede de referência bem definida e regulada para os usuários. Um estudo verificou que, dos 27 estados brasileiros, apenas 63% operacionalizaram os seus Planos Estaduais de Atenção Oncológica, e, destes, a maioria está em desacordo com as diretrizes estabelecidas. Além disso, estados das regiões Sul e Sudeste apresentam maior avanço na elaboração e execução dos planos quando comparados aos estados das regiões Norte e Nordeste ^19^.

Uma alternativa que se mostrou promissora para facilitar o acesso de pacientes nos Estados Unidos foram clínicas satélites de quimioterapia, visando a redução da distância percorrida na obtenção do tratamento ^18^.

O tempo para início do tratamento conforme a legislação (60 dias) ^10^ foi de 80%. Entretanto, mais de 85% dos indivíduos foram diagnosticados em estadiamento avançado ^20^. Na perspectiva do indivíduo, isso representa uma piora da sobrevida, uma vez que, quando diagnosticado em estágios iniciais, a sobrevida em um ano é de 63,5% e de 26,0% em estágios avançados ^20^. Para o sistema de saúde, essa elevada taxa de diagnósticos tardios configura significativo atraso e ineficiência do processo diagnóstico ^21^.

Dentre os fatores que estão associados ao diagnóstico tardio, destacam-se: características da fisiopatologia da doença, entre as quais uma sintomatologia inespecífica, e que só se apresentam em estado avançado ^3^; falhas na formação médica, podendo gerar desafios na interpretação de achados radiológicos ^22^; limitada capacidade instalada e de recursos financeiros e humanos do sistema de saúde, podendo implicar na demora para realização de exames diagnósticos ^4^^,^^20^; e características do indivíduo, como: percepções e crenças, estigma tanto em relação ao tabagismo quanto à pessoa com câncer e receio do diagnóstico, o que pode levar a uma demora na procura por atendimento ^23^. Adicionalmente, no contexto brasileiro, não há uma política nacional de rastreamento, apesar de recomendação internacional ^7^.

Neste estudo, observou-se que o Sul apresentou a maior taxa de câncer de pulmão do país. Entre as possíveis explicações para esse resultado, destacam-se: maior longevidade, hábitos de vida dessa população, com destaque para o tabagismo, e melhor estrutura dos serviços e capacidade diagnóstica. Por conseguinte, observa-se um maior número de diagnósticos, gerando um possível aumento nos registros em bases de dados oficiais ^24^.

Os municípios nos quais a cobertura da atenção básica está abaixo da média nacional têm maiores chances de apresentarem casos registrados acima do 3º quartil. Essa associação inversa justifica-se por o serviço oncológico estar concentrado nos grandes centros, que, em geral, têm baixa cobertura da atenção básica ^25^

Apesar dos números alarmantes de incidência e de mortalidade por câncer de pulmão no mundo ^1^, observa-se redução de casos e de mortes por diversos tipos de câncer nas regiões de maior IDH e maior acesso a serviços e saúde ^14^. Ao mesmo tempo, ocorre um aumento de casos em regiões com piores indicadores sociodemográficos, principalmente de cânceres associados à pobreza, como os de estômago, de colo do útero, de pênis e de cavidade bucal ^26^, o que pode representar o impacto das desigualdades tanto socioeconômicas quanto de acesso aos serviços de saúde ^14^.

Uma parcela relevante dos indivíduos diagnosticados em estadiamento inicial não realizou os tratamentos preconizados de cirurgia e/ou radioterapia. Na região Norte, essa proporção chega a mais da metade dos casos. É sabido que o parque tecnológico e recursos humanos em radioterapia no Brasil são insuficientes ^27^. Em termos de acesso ao tratamento cirúrgico, é prioritário adotar estratégias que favoreçam o acesso dos pacientes aos procedimentos cirúrgicos, melhorar a capacitação profissional, aumentar a segurança desses procedimentos e integrar os centros cirúrgicos às unidades que realizam outras modalidades de tratamento, como quimioterapia e radioterapia, favorecendo a integralidade do cuidado oncológico ^28^.

Provavelmente, o acesso à cirurgia é afetado por fatores como diferenças socioeconômicas, comorbidades, idade avançada e localização geográfica dos serviços ^3^. Quanto aos recursos humanos, ressalta-se que os cirurgiões torácicos estão concentrados nas regiões do centro-sul do país e em grandes cidades ^29^. Portanto, mesmo que o número absoluto fosse adequado, sua distribuição ainda seria desproporcional, visto que municípios de porte médio e regiões densamente povoadas no Centro, Norte e Nordeste do Brasil estão desassistidas ^3^. Isso é preocupante, uma vez que maiores tempos para execução de procedimento cirúrgico como início de tratamento estão diretamente associados à diminuição da sobrevida global no câncer de pulmão ^30^.

Considerando-se os achados acerca dos fatores associados ao acesso ao registro de casos, características do tratamento e da mortalidade por câncer de pulmão, conclui-se que tanto a infraestrutura dos serviços quanto os indicadores socioeconômicos foram relevantes para altas taxas de registro de câncer de pulmão, acesso ao tratamento e maior cobertura de casos em relação aos óbitos. Ademais, áreas com menor infraestrutura em termos de serviços de maior complexidade apresentaram maior probabilidade de não registrar casos, apesar de registrarem óbitos por câncer de pulmão. 

Portanto, os resultados reproduzem o padrão de desigualdades encontradas no país, cuja redução é desafiadora. Já que os serviços de maior complexidade tecnológica estão concentrados em determinadas regiões, os processos de regulação e de contratualização são precípuos para assegurar o acesso integral ao tratamento oncológico. Além dos aspectos relacionados à existência da infraestrutura, é necessário criar e implementar estratégias para a capacitação do profissional em oncologia e para a disseminação das melhores práticas no cuidado oncológico, permitindo a ampliação do acesso oportuno ao diagnóstico e ao tratamento.

Diante do cenário apresentado, sugere-se que estudos futuros abordem o pareamento de dados disponíveis, a análise do itinerário terapêutico do paciente e o impacto da adoção de rastreamento, incluindo estratégias e critérios de elegibilidade.

## Data Availability

O banco de dados utilizados na pesquisa está disponível em: https://data.scielo.org/dataset.xhtml?persistentId=doi:10.48331/scielodata.8HSKF2
